# Antibody Response to Feline Calicivirus Vaccination in Healthy Adult Cats

**DOI:** 10.3390/v11080702

**Published:** 2019-07-31

**Authors:** Michèle Bergmann, Stephanie Speck, Anna Rieger, Uwe Truyen, Katrin Hartmann

**Affiliations:** 1 Clinic of Small Animal Medicine, Centre for Clinical Veterinary Medicine, LMU Munich, 80539 Munich, Germany; 2 Institute of Animal Hygiene and Veterinary Public Health, University of Leipzig, 04103 Leipzig, Germany

**Keywords:** FCV, protection, immunization, antibody titer

## Abstract

This study evaluated the prevalence of feline calicivirus (FCV) antibodies and response to vaccination in healthy adult cats. Cats >1 year (*n* = 111) that had not been vaccinated within 12 months of enrollment in the study received a vaccine containing inactivated FCV antigen strains 431 and G1. Antibodies were determined on Days 0, 7, and 28 by virus neutralization (VN) using FCV isolate KS20, and by broad spectrum blocking FCV enzyme-linked immunosorbent assay (ELISA). Factors associated with the presence of antibodies and vaccine response were determined by uni- and multivariate analysis. Pre-vaccination antibodies were detected in 62.2% of cats (CI_95%_: 52.9–70.1) by VN and in 77.2% (CI_95%_: 67.5–84.6) by ELISA. A ≥4-fold titer increase after vaccination was observed in 13.6% (CI_95%_: 8.3–21.4) of cats with VN and 33.7% (CI_95%_: 24.5–44.5) with ELISA. Factors associated with the presence of pre-vaccination VN antibodies were age (≥2 years; OR: 7.091; *p* = 0.022) and lack of previous vaccination (OR: 3.472; *p* = 0.014). The presence of pre-vaccination ELISA antibodies was associated with time since last vaccination (OR: 5.672; *p* = 0.043). Outdoor cats were more likely to have a ≥4-fold ELISA titer increase (OR: 5.556; *p* = 0.005). Many cats had pre-vaccination FCV antibodies, and their presence depended on previous vaccinations and increases with age. A ≥4-fold titer increase was rarely observed and was influenced by the lifestyle of the cat.

## 1. Introduction

Infection with feline calicivirus (FCV) is common in cats and can result in acute oral and upper respiratory tract disease [1]. Highly virulent strains of FCV can lead to systemic disease with high mortality rates [2,3,4]. Vaccination is therefore strongly recommended for all cats [1]. However, FCV vaccines do not provide complete protection because of the wide spectrum of genetic and antigenic variability of the virus [5], which results in many different field strains with limited cross-reactivity. The protection provided by vaccines is limited to a certain spectrum of field strains, and thus vaccine strains should ideally be adapted to field isolates that currently circulate in the local environment [6,7]. Unfortunately, this is not feasible in veterinary medicine, and current FCV vaccines contain either single vaccine strains (F9 or 255) or a combination of two vaccine strains (G1 and 431). Single vaccine strains have been used for several decades. However, they are discussed as being less effective due to the high mutation rate of FCV [8,9]. One study found that the neutralizing capacity of antibodies after vaccination in Swedish cats was 11.5% (F9) and 64.1% (255) for older single-strain vaccines compared with 70.5% (G1) and 89.7% (431) [8] for recently introduced vaccines containing two strains. In an experimental study, vaccination with a combination of G1 and 431 produced higher titers of neutralizing antibodies, better clinical protection, and reduction of virus shedding after heterologous challenge than vaccination with either of these two strains alone [9]. These results are in contrast to the findings of a recent study in which F9 was shown to be highly effective in neutralizing 97% of field isolates in six different European countries [10].

The immunity afforded by FCV vaccines is considered to be mainly humoral, and FCV antibodies can be used to predict immunity to the respective strains [11]. In adult cats that had been vaccinated, detection of FCV-specific VN titers >8 has been considered predictive of whether cats were susceptible to disease [11]. However, today, there is the consensus that any titer (independent of height) indicates protection in adult cats, because presence of antibodies is indicative of the presence of memory cells that can rapidly boost the cat’s antibody response in the event of infection [1]. Studies in client-owned cats showed that antibodies against FCV were present in 39.2% of cats in Brazil [12], 49.3% of cats in Germany [13], and 92.4% of cats in the United States [11]. Antibodies can be detected by virus neutralization (VN) or enzyme-linked immunosorbent assay (ELISA) [14], but only VN detects antibodies that neutralize infectious particles and prevent infection [15]. However, the results of VN testing greatly depend on the degree of antigenic relationship between the isolate used in the test and the isolate collected from cats that have been vaccinated or had natural infection. ELISA usually use a monoclonal antibody against a conserved epitope of the capsid protein and, thus, are able to detect antibodies against a broad spectrum of FCV strains [16].

So far, no studies have been published on the association between the prevalence of FCV antibodies and vaccination history or on the response of field cats with pre-existing antibody titers to renewed vaccination. The aims of the study were to evaluate: (1) the prevalence of antibodies against FCV in healthy adult cats by means of VN using the FCV isolate KS20 as antigen, and ELISA using FCV p66 antigen; (2) the factors associated with the presence of pre-vaccination antibodies; (3) the response to vaccination with an inactivated FCV vaccine by measuring the antibody response within a 28-day period after vaccination; and (4) the factors associated with response to vaccination.

## 2. Materials and Methods

### 2.1. Study Population 

Serum samples from 111 cats were included in this prospective study. A minimum sample size of at least 96 cats was determined by power analysis, based on an assumed FCV antibody prevalence of 50%, with a 95% confidence interval and a 10% margin of error. The protocol was approved by the ethical committee of the Centre of Clinical Veterinary Medicine of the LMU Munich (reference number 55.2-1-54-2532.3-62-11).

All cats were presented to the Clinic of Small Animal Medicine, Centre for Clinical Veterinary Medicine, LMU Munich, or to an animal shelter in Southern Germany for vaccination. Inclusion criteria for the cats were: (1) unremarkable physical examination; (2) ≥1 year of age; and (3) last vaccination against FCV >1 year ago. Exclusion criteria were: (1) the administration of immunosuppressive drugs or passive immunization within the previous four weeks; and (2) infection with feline immunodeficiency virus (FIV) and/or feline leukemia virus (FeLV) determined by a commercial ELISA (SNAP Kombi Plus FeLV/FIV antibody test^®^, IDEXX GmbH, Ludwigsburg, Germany). The signalment of the cats is shown in Table 1.

### 2.2. Study Protocol

On Day 0, each cat received a single dose of a vaccine containing inactivated FCV antigen strains G1 and 431 with at least 2.0 ELISA units per dose, as well as feline herpesvirus-1 (FHV-1), and feline parvovirus (FPV); response to FHV-1 and FPV vaccination was not part of the present study.

For the detection of pre- and post-vaccination FCV antibodies, serum samples were collected on Days 0, 7, and 28, and frozen at −20 °C until analysis. In 23 cats, a blood sample could not be obtained on Day 7, and in one of these 23 cats, a sample could not be obtained on Day 28. Serum samples from 104 cats were available for detection of antibodies by ELISA. Antibodies could not be measured by ELISA on Day 0 (*n* = 12), Day 7 (*n* = 28), and Day 28 (*n* = 20) in some of the cats because the amount of serum was too small.

Data on signalment (age, breed, sex, neutering status, and body weight), origin (breeder, private household, or animal shelter), environment (urban or rural), housing conditions (multi-cat or single-cat household), lifestyle (indoor or outdoor), cohabitation with dogs, and vaccination status (previous vaccination, complete vaccination series, and time since last vaccination) were collected from the owners on Day 0. In addition to obtaining a detailed history of the cats, a physical examination was carried out on Days 0, 7, and 28. The occurrence of vaccine-associated adverse events (VAAEs) was assessed on Days 7 and 28. 

Most of the cats (72 of 111, 64.9%) had been previously vaccinated against FVC. Vaccination status was unknown in three cats beyond the previous 12 months. Only 20 of 72 (27.8%) cats had received a complete vaccination series against FCV, which was defined as follows: for young kittens, vaccination was started at 6–8 weeks of age and booster vaccinations were given at 3–4 week intervals until approximately 16 weeks of age, and 11 to 13 months later; and, for cats >12 weeks of age, vaccination was considered complete if two vaccinations had been administered 3–4 weeks apart, followed by a vaccine 11–13 months later [17]. To be considered completely vaccinated, booster vaccinations were required at intervals of no greater than three years in cats that had received the primary series.

### 2.3. Detection of Antibodies by VN 

Sera were heat-inactivated for 30 min at 56 °C and then pre-diluted 1:5 with dilution buffer (PBS). On a 96-well plate, 60 μL of PBS were placed in each well. Subsequently, 60 μL of the prediluted serum were pipetted into the wells of the first column of the 96-well plate, mixed gently and titrated to column 12. Positive and negative controls were included. Feline calicivirus isolate KS20 was used for VN. Sixty microliters of FCV (200 median tissue culture infective dose per 0.1 mL) were added to each well and the plate was incubated at 37 °C for 1 h. After incubation, the serum virus suspensions were transferred to a 96-well plate with CRFK cells and incubated for 5–7 days at 37 °C in 5% CO_2_. The cell layers were examined microscopically each day for signs of a cytopathogenic effect indicating virus growth. The final result was read on Day 7. A titer <10 was considered negative. A positive antibody response to vaccination was defined as ≥4-fold titer increase by Day 28 (compared to Day 0).

### 2.4. Detection of Antibodies by ELISA 

Microplates were coated with a capture antibody for 18 h at 5 °C and then rinsed with classical PBS-Tween solution. In separate dilution plates, the test sera and positive and negative controls were incubated with a FCV p66 antigen for 18 h at 5 °C. The serum/antigen mixture was transferred to the ELISA plate coated with the capture antibody. Plates were incubated for 3 h at 37 °C and then rinsed. A broadly cross-reactive anti-FCV p66 conjugated to horseradish peroxidase (HRP) was added, and, after 1 h at 37 °C, plates were rinsed and incubated with HRP substrate for 30 min at 20 °C. The reaction was stopped with 1 N sulfuric acid, and optical density was read at 450–630 nm. The ELISA serum titer corresponded to the inverse of the dilution of the test serum that had an optical density equal to 50% of the maximal optical density. A titer <20 was considered negative. Antibody response to vaccination was defined as ≥4-fold titer increase by Day 28 (compared to Day 0).

### 2.5. Statistical Analysis

Statistical analysis was done with SPSS version 22 (IBM Corporation, Armonk, NY, USA). Fisher’s exact test was used to assess risk factors associated with: (1) the presence of pre-vaccination antibodies; and (2) the response to vaccination. Evaluated risk factors are listed in Table 1 and Table 2. Multivariate logistic regression analysis was carried out for factors significant in univariate analysis with backwards stepwise selection based on Wald. This procedure uses the Wald statistics to remove stepwise the factors with the smallest effect on the diagnostic status variable until only relevant factors are included. Level of significance for all analyses was set at *p* <0.05. A ≥4-fold titer increase by Day 28 (compared to Day 0) was regarded as response to vaccination.

## 3. Results

### 3.1. Prevalence of Pre-Vaccination Antibodies 

In VN, antibody titers ≥10 against strain KS20 on Day 0 were present in 69 of 111 (62.2%; 95% CI, 52.9–70.1) cats (Table 3). One cat was VN-positive but ELISA-negative by Day 0.

In univariate analysis, the factors age, time since last vaccination, vaccination status, and complete vaccination were significantly associated with the occurrence of pre-vaccination VN antibodies. Results of uni- and multivariate factor analysis are shown in Table 1. Only the factors age and vaccination status remained significantly associated with presence of VN antibodies in multivariate analysis. Outdoor cats were more likely to have pre-vaccination VN antibodies than indoor cats (OR: 7.091; *p* = 0.022). Cats that had been previously vaccinated were more likely to have pre-vaccination VN antibodies than cats that had not been vaccinated before enrollment in the study (OR: 3.472; *p* = 0.014).

In ELISA, antibodies were present in 71 of 92 (77.2%; 95% CI, 67.5–84.6) cats before vaccination (Table 3). Sixteen cats were ELISA-positive but VN-negative. 

In univariate analysis, the factors age, neutering status, origin, housing conditions, time since last vaccination, and vaccination status were significantly associated with the presence of pre-vaccination antibodies (Table 1). In multivariate analysis, only the factor time since last vaccination remained significant. Cats that had their last vaccination within 1–3 years were more likely to have pre-vaccination ELISA antibodies than cats that had never been vaccinated (OR: 5.672; *p* = 0.043). 

### 3.2. Titer Increase after Vaccination

**Figure 1:**
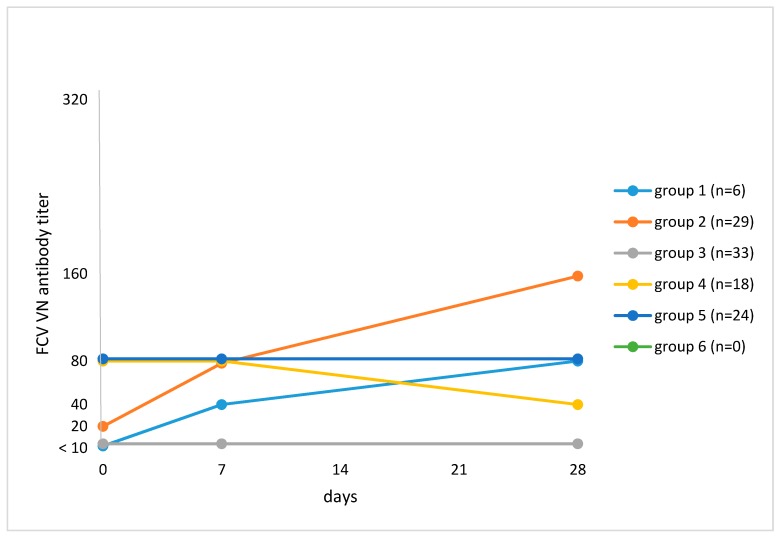
Grouping of cats based on median feline calicivirus (FCV) antibody titers on Day 0 and changes in titer after vaccination against FCV in virus neutralization (VN) testing. The numbers of cats at each time point are given in Section 2.2. Study protocol. Group 1: Titer <10 on Day 0 and subsequent increase in titer (*n* = 6; 5.5%). Median titers: Day 0, <10; Day 7, 40; Day 28, 80. Group 2: Titer ≥10 on Day 0 and subsequent increase in titer (*n* = 29; 26.4%). Median titers: Day 0, 20; Day 7, 80; Day 28, 160. Group 3: Titer <10 pre- and titer <10 post-vaccination (*n* = 33; 30.0%). Group 4: Titer ≥10 on Day 0 and subsequent decrease in titer by Day 28 (*n* = 18; 16.4%). Median titers: Day 0, 80; Day 7, 80; Day 28, 40. Group 5: Titer ≥10 on Day 0 and no change in titer after vaccination (*n* = 24; 21.8%). Median titers: Day 0, 80; Day 7, 80; Day 28, 80. Group 6: Titer ≥10 on Day 0, an increase in titer by Day 7 and a decrease in titer by Day 28 (*n* = 0; 0%).

VN testing revealed a ≥4-fold titer increase after vaccination by Day 28 when compared to Day 0 in 15 of 110 (13.6%; 95% CI, 8.3–21.4) cats (Table 3). One cat had no pre-vaccination VN antibody titer but had a titer of 1280 on Days 7 and 28 after vaccination; this cat was antibody-negative (<20) in ELISA on all days. Four cats had a ≥4-fold titer increase in VN but not in ELISA. Cats were categorized according to their antibody response to vaccination (Figure 1). Cats in Group 1 (*n* = 6; 5.5%) had an antibody titer <10 on Day 0 and had an increase in antibody titer during the study, which was ≥4-fold in four of six cats. In Group 2 (*n* = 29; 26.4%), cats had an antibody titer ≥10 on Day 0 and had an increase in antibody titer after vaccination, which was ≥4-fold in 11 of 29 cats. Cats in Group 3 (*n* = 33; 30.0%) had no titer pre- and post-vaccination (<10). Cats in Group 4 (*n* = 18; 16.4%) had an antibody titer ≥10 on Day 0 and a decrease in titer by Day 28. Group 5 consisted of 24 cats (21.8%) that had an antibody titer ≥10 on Day 0, which remained unchanged for the remainder of the study. There were no cats with a pre-vaccination antibody titer, an increase in titer by Day 7, and a decrease by Day 28 (Group 6). Univariate analysis failed to identify factors that were associated with a ≥4-fold increase in titer (Table 2).

**Figure 2:**
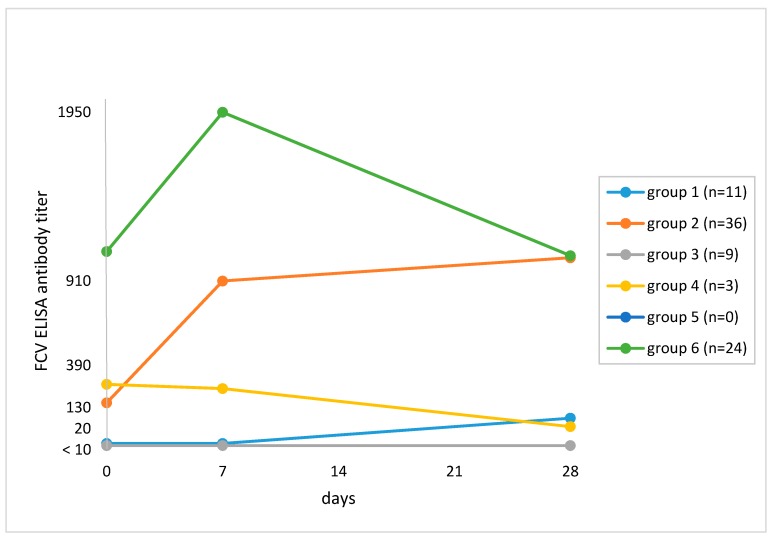
Grouping of cats based on median feline calicivirus (FCV) antibody titers on Day 0 and changes in titer after vaccination against FCV in ELISA. The numbers of cats at each time point are given in Section 2.2. Study protocol. Group 1: Titer <20 on Day 0 and subsequent increase in titer (*n* = 11; 13.3%). Median titers: Day 0, <20; Day 7, <20; Day 28, 60. Group 2: Titer ≥20 on Day 0 and subsequent increase in titer (*n* = 36; 43.4%). Median titers: Day 0, 150; Day 7, 855; Day 28, 1020. Group 3: Titer <20 pre- and titer <20 post-vaccination (*n* = 9; 7.5%). Median titers: Day 0, <20; Day 7, <20; Day 28, 20. Group 4: Titer ≥20 on Day 0 and decrease in titer by Day 28 (*n* = 3; 3.6%). Median titers: Day 0, 265; Day 7, 235; Day 28, 30. Group 5: Cats with pre-vaccination antibody titer ≥20 on Day 0 and no change in titer after vaccination (*n* = 0; 0%). Group 6: Titer ≥20 on Day 0 and decrease in titer by Day 28 (*n* = 24; 28.9%). Median titers: Day 0, 1125; Day 7, 1960; Day 28, 1050.

The ELISA revealed a ≥4-fold titer increase after vaccination by Day 28 when compared to Day 0 in 28 of 83 (33.7%; 95% CI, 24.5–44.5) cats (Table 3). Twenty-two cats had a ≥4-fold increase in titer using ELISA but not VN testing. Figure 2 shows the antibody responses of the cats. Cats in Group 1 (*n* = 11; 13.3%) had an antibody titer <20 on Day 0 and a subsequent increase, which was ≥4-fold in 4 of 11 cats. In Group 2 (*n* = 36; 43.4%), cats had an antibody titer ≥20 on Day 0 and a subsequent increase, which was ≥4-fold in 24 of 36 cats. Cats in Group 3 (*n* = 9; 7.5%) had an antibody titer <20 on Day 0 and no subsequent increase; six of these cats had no antibodies pre- and post-vaccination. Cats in Group 4 (*n* = 3; 3.6%) had a decrease in titer by Day 28. There were no cats with a pre-vaccination antibody titer and no subsequent increase in titer (Group 5). Cats in Group 6 (*n* = 24, 28.9%) had a pre-vaccination antibody titer ≥20, an increase in titer by Day 7 and a decrease by Day 28. 

Univariate analysis showed that the factor lifestyle was associated with a ≥4-fold increase in titer. Outdoor cats were more likely to have a ≥4-fold increase in titer in ELISA (OR: 5.556; 95% CI, 1.671–18.47; *p* = 0.005) (Table 2).

## 4. Discussion

In the present study, antibody titers pre- and post-vaccination were measured using two different methods, VN and ELISA. Feline calicivirus isolate KS20 used in VN was shown to have cross-reactivity with sera from cats originating from the same geographical region as the cats in the present study and was therefore chosen [18]. Virus neutralization was performed in this study because it detects antibodies that neutralize infectious particles and thus prevent infection [15]; however, VN is thought to be less sensitive than ELISA because the results are strongly influenced by the degree of antigenic relationship between the isolate used in the test and the isolate obtained from previously vaccinated or infected cats. Isolates from the cats were unknown, which might explain why VN was less often positive compared with ELISA in the present study. ELISA was used in addition to VN because it has been shown to have a broad spectrum and detects antibodies produced after vaccination with the G1 and 431 vaccine strains [14], which are commonly used for FCV vaccination in Germany. On Day 0, ELISA had more positive tests results than VN confirming its broader spectrum. Comparison of VN and ELISA results was of particular interest in relation to the response to vaccination because the cats were vaccinated with a vaccine containing strains G1 and 431. Virus neutralization had fewer positive results after vaccination than ELISA, suggesting that VN does not always detect antibodies against vaccine strains G1 and 431 [9].

Prevalence of pre-vaccination antibodies was 62.2% in VN testing and 77.2% in ELISA. A study in France showed that 86.5% of unowned cats in a rural area had FCV antibodies using the same ELISA as used in the present study [19]. In Brazil, 39.2% of cats had antibodies against FCV isolate SV 65/90 [12], and in the United States, 92.4% of cats had antibodies against FCV isolate F9 [11]. An association between prevalence of antibodies and the previous vaccination regime was likely in the above-mentioned studies but statistical analysis was not performed. A study of 347 cats taken into an animal shelter in Florida found that cats older than six months (OR: 11.0) and cats from private households (OR: 2.1) were more likely to have FCV antibodies but the vaccination histories were not available [20]. 

One aim of the present study was to evaluate the association of possible factors with the presence of pre-vaccination antibody titers. The association between presence of VN antibodies and vaccination status (vaccinated or not vaccinated previously) was an interesting finding and was also seen in cats tested for antibodies against feline panleukopenia after MLV vaccination [21]. The factor time since last vaccination was not associated with the occurrence of pre-vaccination VN antibodies. This is in agreement with the results of a study by Mouzin and coworkers (2004), in which the geometric mean of the titer against FCV after MLV vaccination did not decrease when time since last vaccination increased [22]. However, time since last vaccination was correlated with the presence of ELISA antibodies in the present study and cats that had been vaccinated within the last 1–3 years were more likely to have pre-vaccination antibodies than cats that had not been vaccinated. The type of vaccine can also affect the antibody response, and MLV vaccines are known to elicit strong and long-lasting antibody responses [23]. Twelve of 35 cats that had never been vaccinated were antibody-positive in both VN and ELISA. Except for one, all of these cats lived (*n* = 9) or had previously lived (*n* = 2) in multi-cat households at the time of study, and therefore natural exposure was the most likely reason for the antibodies. 

Multivariate analysis did not identify any environmental factors that had significant associations with the prevalence of antibodies. This was surprising because it is generally assumed that cats living in multi-cat environments and/or cats with outdoor access are more likely to undergo natural exposure to FCV and subsequent more commonly have antibodies. Another study also failed to detect an association between environmental factors and presence of FCV antibodies [22]. 

In the present study, cats ≥2 years were more likely to have pre-vaccination VN antibodies than younger cats. Older cats are more likely to have been exposed to the virus and are therefore more likely to have antibodies against FCV [24]. In cats <2 years of age, interference with maternally-derived antibodies during the primary vaccination series is another possible reason for vaccination failure and lack of antibodies [25].

An increase in VN antibody titer after vaccination was seen in only 31.8% of the cats compared with 56.6% of the cats that had an increase in ELISA antibody titer. A likely reason for this is that VN did not reliably detect antibodies against FCV strains G1 and 431. Another possibility is that neutralizing antibodies occurred only after the 28-day study period. Likewise, a ≥4-fold titer increase was less commonly identified using VN (13.6% of cats) compared with ELISA (33.7%). The use of an inactivated vaccine could be the reason for the low number of cats with a ≥4-fold increase in titer in the present study [23].

Five cats had negative ELISA (<20) and VN (<10) test results throughout the entire study and could therefore be considered non-responders. It is possible that these cats developed a cellular response in the absence of a humoral response. Protection against FCV in the absence of neutralizing antibodies 1–4 weeks after vaccination has been reported in experimental challenge studies [9,26]. A non-response to vaccination might occur because the immune system fails to recognize the vaccine antigen [27]. Other reasons for a poor response to vaccination including concurrent and chronic disease, immunosuppression, and faulty vaccine administration and storage were ruled out.

One cat that lived in a multi-cat household had no increase in ELISA titers but had a strong increase in VN titers (<10 on Day 0; 1280 on Days 7 and 28). This could be explained by an incidental natural infection with a strain not cross-reactive with strains G1 and 431.

Some cats had pre-existing antibodies in VN and ELISA and a subsequent decrease in titer after vaccination. This could have been the result of binding of pre-existing antibodies to the vaccine virus. Examination of the course of antibody titer in these cats would require a longer study period than the 28-day period used. 

There are no studies so far evaluating antibody response in cats with different pre-vaccination antibody titers. Outdoor cats were more likely to have a ≥4-fold increase in ELISA than cats kept indoors. Outdoor cats are more likely to have been exposed to FCV, and memory cells generated during the primary vaccination response tend to be more effective at producing antibodies in response to re-vaccination or field-virus re-exposure [23]. 

Interestingly, the pre-vaccination antibody titer was not associated with an increase in titer after vaccination. It has been proposed that high antibody levels neutralize the vaccine virus before it stimulates the immune system, which has been shown in FPV vaccination [28]; however, this cannot be confirmed for FCV based on the results of the present study. Many veterinarians today choose to measure parvovirus antibody titers to determine whether adult cats and dogs require re-vaccination [28,29,30]. A semi-quantitative in-house test for the detection of FCV, FPV, and FHV-1 antibodies is available for use in practice in several countries. It can be useful for determining whether vaccination is required at the time of an individual health care assessment, although the benefit of measuring FCV antibodies before vaccination is still discussed controversially. There was no association between lack of pre-vaccination antibodies and a ≥4-fold titer increase after vaccination in the present study. In addition, and even more importantly, the value of measuring FCV antibodies to predict protection is generally limited as antibodies detected in a cat do not necessarily protect against the strains in the field [1]. 

One limitation of the study was the small amount of available serum (especially for ELISA), which meant that FCV antibodies could not be determined in all cats. In addition, it should be noted that a lack of an increase in antibody titer is not equivalent to a lack of protection against disease as cell-mediated immunity can also be an effective mode of protection [23]. The vaccine used in the present study has been shown to provide protection against challenge even in the absence of detectable neutralizing antibodies [9]. 

## 5. Conclusions

Many cats had pre-vaccination antibodies against FCV even those that had been vaccinated more than one year before the start of the study. The prevalence of antibodies depended on the vaccination history and age of the cats. A ≥4-fold titer increase after vaccination was rare and not associated with the pre-vaccination antibody titer of the cat. Considering the results of the present study and the fact that different FCV strains circulate in the cat population, measuring the presence of FCV antibodies cannot replace routine vaccination against FCV in cats. 

## Figures and Tables

**Table 1:** Characteristics of cats and association with presence of pre-vaccination VN antibodies and ELISA antibodies to feline calicivirus in uni- and multivariate analysis.

Variable	Category	Number of Cats	Cats with Pre-Vaccination FCV Antibodies in VN	Univariate Analysis VN	Multivariate Analysis VN			Number of Cats	Cats with Pre-Vaccination FCV Antibodies in ELISA	Univariate Analysis ELISA	Multivariate Analysis ELISA		
				*p*	*p*	95% CI	OR			*p*	*p*	95% CI	Odds Ratio
Age	1 > 2 years	17	2/17	<0.001 ^a^	Ref. ^c^	NA ^d^	NA ^d^	14	4/14	<0.001 ^a^	Ref. ^c^	NA ^d^	NA ^d^
	≥2 years	94	67/94		0.022 ^a^	1.327–37.904 ^a^	7.091 ^a^	78	67/78		0.061	-	-
Breed	DSH	37	25/37	0.534	-			30	23/30	1.000	-		
	Purebred	74	44/74					62	48/62				
Sex	Female	60	37/60	1.000	-			47	37/47	0.806	-		
	Male	51	32/51					45	34/45				
Weight	<2 kg	15	7/15	0.171	-			13	11/13	0.067	-		
	2–4 kg	42	23/42					34	21/34				
	4–6 kg	48	34/48					40	34/40				
	>6 kg	6	5/6					5	5/5				
Neutering status	Intact	32	16/32	0.130	-			27	17/27	0.013 ^a^	eliminated ^b^		
	Neutered	97	53/97					65	55/65				
Origin	Private	32	32/56	0.567	-			48	33/48	0.011 ^a^	Ref. ^c^	NA ^d^	NA ^d^
	Shelter	35	23/35					27	26/27		0.998	-	-
	Breeder	20	14/20					17	12/17		0.419	-	-
Environment	Urban	89	55/89	1.000	-			75	57/75	0.753	-		
	Rural	22	14/22					17	14/17				
Lifestyle	Indoor	91	54/91	0.215	-			76	56/76	0.107	-		
	Outdoor	20	15/20					16	15/16				
Cohabitation with dogs	Yes	28	17/28	1.000	-			25	20/25	0.786	-		
	No	83	52/83					67	51/67				
Housing conditions	Multi-cat household	92	54/92	0.123	-			76	55/76	0.018 ^a^	eliminated ^b^		
	Single-cat household	19	17/19					16	16/16				
Time since last vaccination	Never	39	14/39	<0.001 ^a^	eliminated ^b^			30	15/30	<0.001 ^a^	Ref. ^c^	NA ^d^	NA ^d^
	>3 years	13	9/13					13	11/13		0.135	-	-
	1–3 years	59	46/59					49	45/49		0.043 ^a^	1.052–30.575 ^a^	5.672 ^a^
Vaccination status *	Vaccinated	72	55/72	<0.001^a^	0.014 ^a^	0.935–1.284 ^a^	3.472 ^a^	62	56/62	<0.001 ^a^	eliminated ^b^		
	Not vaccinated	35	12/35		Ref. ^c^	NA ^d^	NA ^d^	26	12/26				
Complete vaccination series	Yes	20	17/20	0.022 ^a^	eliminated ^b^			17	16/17	0.106	-		
	No	91	52/91					75	55/75				

* Vaccination status was unknown in four cats. ^a^ Values indicate statistical significance. ^b^ The factor was eliminated by the variable selection process of the logistic regression model and was thus not associated with the presence of pre-vaccination antibodies. ^c^ Ref.; reference value indicating that this category was used as baseline for comparison for each variable. ^d^ NA, not applicable; OR, odds ratio; FCV, feline calicivirus; *p*, *p*-value; CI, confidence interval; VN, virus neutralization.

**Table 2:** Factors associated with ≥4-fold antibody titer increase in VN and ELISA after vaccination against feline calicivirus by Day 28 when compared to Day 0.

Variable	Category	Number of Cats	Cats with ≥4-Fold FCV Titer Increase in VN by Day 28	Univariate Analysis with the Results of the VN	Number of Cats	Cats with ≥4-Fold FCV Titer Increase in ELISA by Day 28	Univariate Analysis with the Results of the ELISA
				*p*			*p*
Age	1 > 2 years	16	1/16	0.693	13	2/13	0.202
	≥2 years	94	14/94		70	26/70	
Breed	DSH	73	11/73	0.770	56	23/56	0.050
	Purebred	37	4/37		27	5/27	
Sex	Female	60	9/60	0.783	44	14/44	0.817
	Male	50	6/50		39	14/39	
Weight	<2 kg	15	2/15	1.000	12	2/12	0.472
	2–4 kg	41	6/41		31	10/31	
	4–6 kg	48	7/48		36	14/36	
	>6 kg	6	0/6		4	2/4	
Neutering status	Intact	31	1/31	0.063	25	6/25	0.312
	Neutered	79	14/79		58	22/58	
Origin	Breeder	20	1/20	0.369	15	2/15	0.183
	Shelter	34	4/34		22	9/22	
	Private	56	10/56		46	17/46	
Environment	Urban	89	11/89	0.480	70	25/70	0.528
	Rural	21	4/21		13	3/13	
Lifestyle	Indoor cat	90	12/90	1.000	68	18/68	0.005 ^a^
	Outdoor cat	20	3/20		15	10/15	
Cohabitation with dogs	Yes	28	5/28	0.525	21	8/21	0.790
	No	82	10/82		62	20/62	
Housing conditions	Multi-cat household	91	13/91	1.000	67	23/67	1.000
	Single-cat household	19	2/19		16	5/16	
Time since last vaccination	1–3 years	59	5/59	0.207	45	18/45	0.499
	>3 years	13	2/13		12	3/12	
	Never	38	8/38		26	7/26	
Vaccination status *	Vaccinated	72	7/72	0.080	57	21/57	0.292
	Not vaccinated	35	8/35		23	5/23	
Complete vaccination series	Yes	20	2/20	1.000	16	5/16	1.000
	No	90	13/90		67	23/67	
Pre-vaccination antibodies	Yes	41	4/41	0.407	63	23/63	0.423
	No	69	11/69		20	5/20	

* Vaccination status was unknown in four cats. ^a^ Value indicates statistical significance. FCV, feline calicivirus; *p*, *p*-value; CI, confidence interval; VN, virus neutralization.

**Table 3:** Comparison of the results of enzyme-linked immunosorbent assay (ELISA) and virus neutralization (VN) testing.

	VN Antibodies Against FCV			ELISA Antibodies Against FCV		
	Day 0	Day 28	≥4-Fold Increase by Day 28	Day 0	Day 28	≥4-Fold Increase by Day 28
**positive**	69/111 (62.2%)	75/110 (68.2%)	15/110 (13.6%)	71/92 (77.2%)	78/84 (92.9%)	28/83 * (33.7%)
**negative**	42/111 (37.8%)	35/110 (31.8%)	95/110 (86.4%)	21/92 (22.8%)	6/84 (7.1%)	55/83 (66.3%)

* In one cat, blood samples were not available on Day 0. VN, virus neutralization; FCV, feline calicivirus
